# Evaluation of Gene Expression and Alginate Production in Response to Oxygen Transfer in Continuous Culture of *Azotobacter vinelandii*

**DOI:** 10.1371/journal.pone.0105993

**Published:** 2014-08-27

**Authors:** Alvaro Díaz-Barrera, Fabiola Martínez, Felipe Guevara Pezoa, Fernando Acevedo

**Affiliations:** Escuela de Ingeniería Bioquímica, Pontificia Universidad Catolica de Valparaiso, Valparaiso, Chile; Naval Research Laboratory, United States of America

## Abstract

Alginates are polysaccharides used as food additives and encapsulation agents in biotechnology, and their functional properties depend on its molecular weight. In this study, different steady-states in continuous cultures of *A*. *vinelandii* were established to determine the effect of the dilution rate (D) and the agitation rate on alginate production and expression of genes involved in alginate polymerization and depolymerization. Both, the agitation and dilution rates, determined the partitioning of the carbon utilization from sucrose into alginate and CO_2_ under oxygen-limiting conditions. A low D (0.07 h^−1^) and 500 rpm resulted in the highest carbon utilization into alginate (25%). Quantitative real-time polymerase chain reaction was used to determine the transcription level of six genes involved in alginate polymerization and depolymerization. In chemostat cultures at 0.07 h^−1^, the gene expression was affected by changes in the agitation rate. By increasing the agitation rate from 400 to 600 rpm, the *algE7* gene expression decreased tenfold, whereas *alyA1*, *algL* and *alyA2* gene expression increased between 1.5 and 2.8 times under similar conditions evaluated. Chemostat at 0.07 h^−1^ showed a highest alginate molecular weight (580 kDa) at 500 rpm whereas similar molecular weights (480 kDa) were obtained at 400 and 600 rpm. The highest molecular weight was not explained by changes in the expression of *alg8* and *alg44* (genes involved in alginate polymerization). Nonetheless, a different expression pattern observed for lyases could explain the highest alginate molecular weight obtained. Overall, the results suggest that the control of alginate molecular weight in *A*. *vinelandii* cells growing in continuous mode is determined by a balance between the gene expression of intracellular and extracellular lyases in response to oxygen availability. These findings better our understanding of the biosynthesis of bacterial alginate and help us progress toward obtain tailor-made alginates.

## Introduction

Alginate is a copolymer of (1–4)-linked residues of β-D-mannuronic acid (M) and α-L-guluronic acid (G) and produced by *Pseudomonas* and *Azotobacter* species [1]. These polymers have a wide range of applications: they are used as food additives and encapsulation agents in biotechnology and have a promising potential for the biomedical field [2]. *Azotobacter vinelandii* is an aerobic bacterium that produces two polymers of industrial interest: poly-β-hydroxybutyrate (PHB) and alginate. Their functional properties depend on their monomer composition and molecular weight [3].

Regarding alginate biosynthesis, it is well known that alginate is synthesized as a polymannuronate from its cytosolic precursor (GDP-M). Nevertheless, the mechanisms involved in the polymerization, modification (acetylation and depolymerization) and translocation steps are poorly elucidated [4]. The alginate depolymerization steps in *A*. *vinelandii* are complex because this microorganism possesses three intracellular lyases (AlgL, AlyA1 and AlyA2) and two extracellular lyases (AlgE7 and AlyA3) [5]. The polymerization process is catalyzed by Alg8, which is a bottleneck in the biosynthesis of alginate [6]. Alg44 has been postulated to play an indirect role in alginate polymerization, facilitating the transport, modification and secretion of alginate [4]. However, the specific role of Alg44 remains unclear.

In cultures operated in continuous, it has been demonstrated that the oxygen transfer rate (OTR), and the dilution rate (D) affect alginate production, particularly the molecular weight of the final product [7]–[9]. By varying the agitation rate and increasing the specific oxygen uptake rate from 2.2 to 4.8 mmol g^−1 ^h^−1^, alginate production can be improved. This improvement can be attributed to changes in carbon flux [9]. A previous study [9] reported that a lower alginate molecular weight can be obtained by increasing the specific oxygen uptake rate; increased expression of *algL* (approximately 8-fold) was hypothesized to result in a decreased molecular weight. Similarly, Díaz-Barrera *et al*. [10] showed that increased alginate molecular weight produced by *A. vinelandii* continuous cultures can be linked to higher *alg8* gene expression.

Recently, Flores *et al*. [11] evaluated the expression of genes involved in alginate polymerization and depolymerization, as well as lyase activity in batch cultures of *A*. *vinelandii* under dissolved oxygen tension (DOT) controlled conditions. These authors showed that in batch cultures at 1% DOT, low lyase activity and high expression levels of *alg8* and *alg44* might be mechanisms by which oxygen regulates the synthesis of alginates. Those experiments were conducted in batch-mode, in which the specific growth rate changes continuously with time, particularly under oxygen-limited conditions. Because the specific growth rate plays an important role in determining the molecular weight of the alginate [8], [12] in this work, different steady-states in continuous cultures were established to evaluate the effect of oxygen supply conditions on alginate production and gene expression at a constant specific growth rate. To extend the knowledge about the effects of oxygen availability and D on alginate production and expression of genes involved in alginate polymerization and depolymerization, the objective of this study was to evaluate how alginate production (particularly its molecular weight) and the expression of genes involved in polymerization (*alg8, alg44*) and depolymerization (*algL*, *algE7*, *alyA1*, and *alyA2*) are influenced by oxygen transfer in continuous cultures of *A*. *vinelandii*.

## Materials and Methods

### Strain and culture medium

Strain ATCC 9046 of *Azotobacter vinelandii* was used. The bacterium was grown under nitrogen fixation conditions. The culture medium used for chemostat cultures was (in g l^−1^): 10 sucrose, 0.66 K_2_HPO_4_, 0.16 KH_2_PO_4_, 0.056 CaSO_4_•2H_2_O, 0.2 NaCl, 0.2 MgSO_4_•7H_2_O, 0.0029 Na_2_MoO_4_•2H_2_O, and 0.027 FeSO_4_•7H_2_O. The sucrose, K_2_HPO_4_ and KH_2_PO_4_ were dissolved in bioreactor and autoclaving at 121°C during 20 min. CaSO_4_•2H_2_O was sterilized separately in the autoclave (121°C, 20 min). To avoid precipitation, the solutions of NaCl, MgSO_4_·7H_2_O, Na_2_MoO_4_·2H_2_O, and FeSO_4_·7H_2_O were separated from the other medium components during sterilization (autoclave at 121°C for 20 min).

### Inoculum preparation

The inoculum for the bioreactor was prepared in 500 ml Erlenmeyer flasks with 100 ml of culture medium, as described previously [10]. The initial pH was adjusted to 7.0 using 2 M NaOH. The microorganism was incubated at 200 rpm and 30°C in an orbital incubator shaker (New Brunswick, USA). After 15 h, the cells were transferred (10% v/v) to a bioreactor operated in batch mode.

### Fermentation conditions

Chemostat experiments operated at steady-state were conducted in a 3 l bioreactor (Applikon, Schiedam, Netherlands) with a working volume of 2 l at 30°C and pH 7.0 controlled by automatic addition of 2 M NaOH. The stirred fermenter was aerated at 2 l min^−1^ (1.0 vvm) and agitated at 400, 500, and 600 rpm. The DOT was measured with a polarographic oxygen probe (Ingold, Mettler-Toledo) and was not controlled. The bioreactor was operated in batch mode for the first 15 h, then in continuous culture mode with D values of 0.07, 0.09 and 0.11 h^−1^. The working volume was kept constant by withdrawing culture broth through a continuously operated peristaltic pump (Cole-Parmer, USA). The continuous culture reached steady state conditions when the optical density at 540 nm (OD_540_) and the sucrose concentration remained constant (<10% variation) after at least 4 residence times.

Samples of cultures (20 ml) were withdrawn from the bioreactor for analytical measurements. All analyses were carried out in triplicate. The results shown are the mean value of two independent chemostat runs, and the standard deviations among replicates are given.

### Analytical methods

Cell growth was estimated gravimetrically, as described previously by Díaz-Barrera *et al*. [10]. Sucrose concentration was determined by hydrolysis with β-fructofuranosidase, followed by the determination of reducing sugars with dinitrosalicylic acid (DNS) reagent [13]. PHB was extracted from cells and quantified by HPLC as crotonic acid. PHB was hydrolyzed to crotonic acid using concentrated H_2_SO_4_: 3 mg of biomass (previously dried) was weighed in an Eppendorf tube; 1 ml of H_2_SO_4_ was added, and the tube heated at 90°C and agitated for 1 h. The sample was diluted with Milli-Q water and was assayed using an HPLC-UV system with an Aminex HPX-87H ion-exclusion organic acid column. Elution was performed with 0.005 M H_2_SO_4_ at 0.6 ml min^−1^ and 35°C [14]. The alginate concentration was quantified by the metahydroxidiphenyl method for the measurement of uronic acid (monomers of alginate) [15]. The molecular weight of alginate was determined by gel permeation chromatography (GPC) using a serial set of Ultrahydrogel columns (UG 500 and Linear Waters) in an HPLC system with a differential refractometer detector (PerkinElmer, USA). Elution was performed with 0.1 M NaNO_3_ at 35°C at a flow rate of 0.8 ml min^−1^ using pullulans from *Aureobasidium pullulans* as molecular weight standards [16].

### Gas analysis and respiratory measurements

Gas analysis was performed by online measurements of O_2_ and CO_2_ in the exit gas and compared with measurements taken of the inlet gas with a gas analyzer (Teledyne Instruments, model 7500). The OTR and carbon dioxide transfer rate (CTR) were determined by gas analysis and calculated by carrying out gas mass balances [17] as follows:
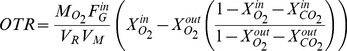
(1)

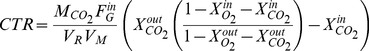
(2)where M_o2_, M_co2_ are the molecular mass of oxygen and carbon dioxide (g mmol^−1^), respectively, F_G_^in^ the volumetric inlet air flow at standard conditions (l h^−1^), V_R_ the working volume (l), V_M_ the mol volume of the ideal gas at standard conditions (l mmol^−1^), X_o2_^in^ and X_co2_^in^ the molar fractions of oxygen and carbon dioxide in the inlet air, respectively (mol mol^−1^), X_o2_°^ut^ and X_co2_°^ut^ the molar fractions of oxygen and carbon dioxide in the outlet air of the fermenter, respectively (mol mol^−1^).

### Quantitative real-time PCR assay

Expression of genes *algL*, *alyA1, alyA2*, *algE7, alg8 and alg44* were analyzed by quantitative RT-PCR. Measurements were carried out in a Light Cycler 2.0 thermocycler (Roche), using commercial Maxima Sybr Green qPCR Master Mix (Thermo Scientific). Fifty micrograms of RNA purified with an RNA Isolation Kit (Roche) and treated with DNAse (Thermo Scientific) was mixed with Revert Aid H minus Reverse Transcriptase (Thermo Scientific) according to the manufacturer’s protocol to obtain the cDNA. The samples were subjected to an initial denaturation at 95°C for 10 min, followed by 40 cycles of 15 s at 95°C and 60 s at 60°C. As an internal standard and control, the expression level of *gyrA* was also determined. Relative gene expression values were obtained using the ΔΔCt method. Table 1 shows the primers used for these analyses. The level of genes expression was normalized according to the level of the *gyrA* mRNA and the data are presented as fold changes of mRNA levels respect of calibrator value (500 rpm).

**Table 1:** Primers used for gene expression analysis by quantitative real-time PCR assay.

Gene	Primers
*alg8*	5′-TGTTGAACCAGCTCTGGAAG-3′
	5′-CCTACCCGCTGATCCTCTAC-3′
*alg44*	5′-CGACAACTTCACCGAAGGG-3′
	5′-TGACGAAGTAGAGGTCGTAGAG-3′
*algE7*	5′-AGATAGGTGCGGTTGGTTTC-3′
	5′-CTCCGACCTGATTCTCGATT-3′
*algL*	5′-GCCCAGTAGGAGTGGTTGTT-3′
	5′-CTGAAATTCTCCAGTTCGCA-3′
*alyA1*	5′-CGGTCGGTATTGCACATAGA-3′
	5′-CAAGATCCACCGTTTGAGTG-3′
*alyA2*	5′-AACTGAGTGCAACCTTGACG-3′
	5′-GCTGCACGTTGATCTTGAAT-3′
*gyrA*	5′-ACCTGATCACCGAGGAAGAG-3′
	5′-AGGTGCTCGACGTAATCCTC-3′

### Calculation of the specific uptake/production rates and carbon recovery

The specific alginate production rate (q_p_), specific sucrose uptake rate (q_s_), specific oxygen uptake rate (q_o2_), and specific carbon dioxide rate (q_co2_) were calculated at steady state conditions, considering D, alginate concentration (P), OTR and CTR values, biomass concentration (X), residual sucrose concentration (S) in the bioreactor and sucrose concentration in the feed medium (S_o_) using the following equations:
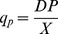
(3)

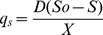
(4)

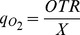
(5)

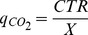
(6)


Carbon recovery under steady state conditions was determined from reactor mass balances according to Díaz-Barrera *et al*. [9], as described previously.

## Results and Discussion

### Continuous cultures at different agitation and dilution rates

**Figure 1:**
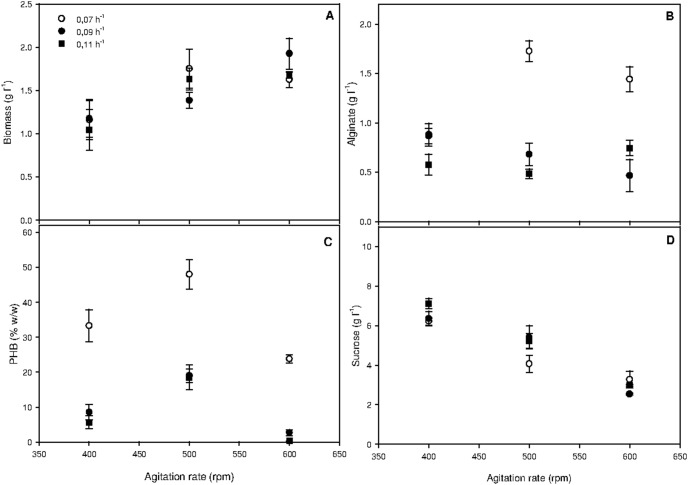
Effect of the agitation rate on biomass, alginate, PHB and sucrose levels in chemostat cultures of *A. vinelandii* conducted at different dilution rates. Data are reported as the mean value ± standard deviation.

Figure 1 shows the effect of agitation rate and D on biomass, sucrose, alginate and PHB concentration at steady state. In a chemostat agitated at 400 rpm, the biomass concentration was similar for the different dilution rates evaluated, reaching approximately 1.0 g l^−1^ (Fig. 1a). An increase in the agitation rate from 500 to 600 rpm with D = 0.09 h^−1^ improved biomass production from 1.3 to 1.9 g l^−1^. It is interesting to note that in chemostat cultures with D = 0.07 h^−1^ and 0.11 h^−1^, increasing the agitation rate from 500 to 600 rpm did not enhance the biomass concentration. In light of these results, the specific growth rate (i.e., D in chemostats) has more influence than the agitation rate (at least between 500 and 600 rpm) on the diversion of carbon into biomass production.

As shown in Fig. 1b, the alginate production at steady state is strongly affected by D. At the lowest D tested (0.07 h^−1^) and 500 rpm, the alginate concentration was 2.5 and 3.5 times higher compared to alginate concentrations obtained at 0.09 and 0.11 h^−1^, respectively. In agreement with this evidence, Díaz-Barrera *et al*. [8] demonstrated that a decrease in the D from 0.08 to 0.05 h^−1^ increased alginate production, whereas biomass and sucrose concentrations remained unchanged.

At a D of 0.09 h^−1^, the alginate concentration decreased from 0.9 to 0.5 g l^−1^ when the agitation rate was increased from 400 to 600 rpm whereas, at a D of 0.11 h^−1^, the alginate concentration was only slightly affected by the agitation rate (Fig. 1b). These data indicate that both the specific growth rate and agitation rate influence alginate production. Under the conditions evaluated, lower D and 500 rpm are the optimal operating conditions for the continuous production of alginate.

Behavior similar to that of alginate production was observed for PHB accumulation. The data show that a lower D (i.e., 0.07 h^−1^) improved PHB production, reaching 48% (w/w) of the dry cell weight for an agitation rate of 500 rpm (Fig. 1c). Regardless of the agitation rate, chemostat cultures conducted at higher D (0.09 and 0.11 h^−1^) showed a decrease in PHB accumulation (less than 19%), which suggests that D affects intracellular PHB content.

Regardless of the dilution rate, an increase in agitation rate decreased the sucrose concentration at steady-state (Fig. 1d), reaching between 2.5 g l^−1^ and 3.3 g l^−1^ depending on the agitation rate of the culture. Considering that K_s_ (saturation constant) values are lower than 0.1 g l^−1^ in sucrose-limited chemostat cultures of *A. vinelandii* [18], our chemostat cultures were not limited by sucrose. Under all the conditions evaluated, the DOT at steady-state was nearly zero, which indicates that the cultures were oxygen-limited. Similar behavior has been observed previously [9].

In the chemostat agitated at 600 rpm, a 1.4 to 1.7-fold increase (depending on D) in sucrose consumption (i.e., inlet sucrose minus sucrose in steady-state) was identified compared to sucrose consumption at 500 rpm. As has been mentioned, in chemostats with D = 0.07 h^−1^ and 0.11 h^−1^, a change in the agitation rate from 500 to 600 rpm did not increase the biomass and alginate concentrations obtained at steady-state; therefore, this increase in sucrose consumption could be related to increased CO_2_ production. To validate this hypothesis, the respiratory activities, i.e., the OTR, CTR, q_o2_ and q_co2_ values of *A*. *vinelandii* cultures were characterized in all of the chemostat cultures.

### Respiratory activities in continuous cultures

**Figure 2:**
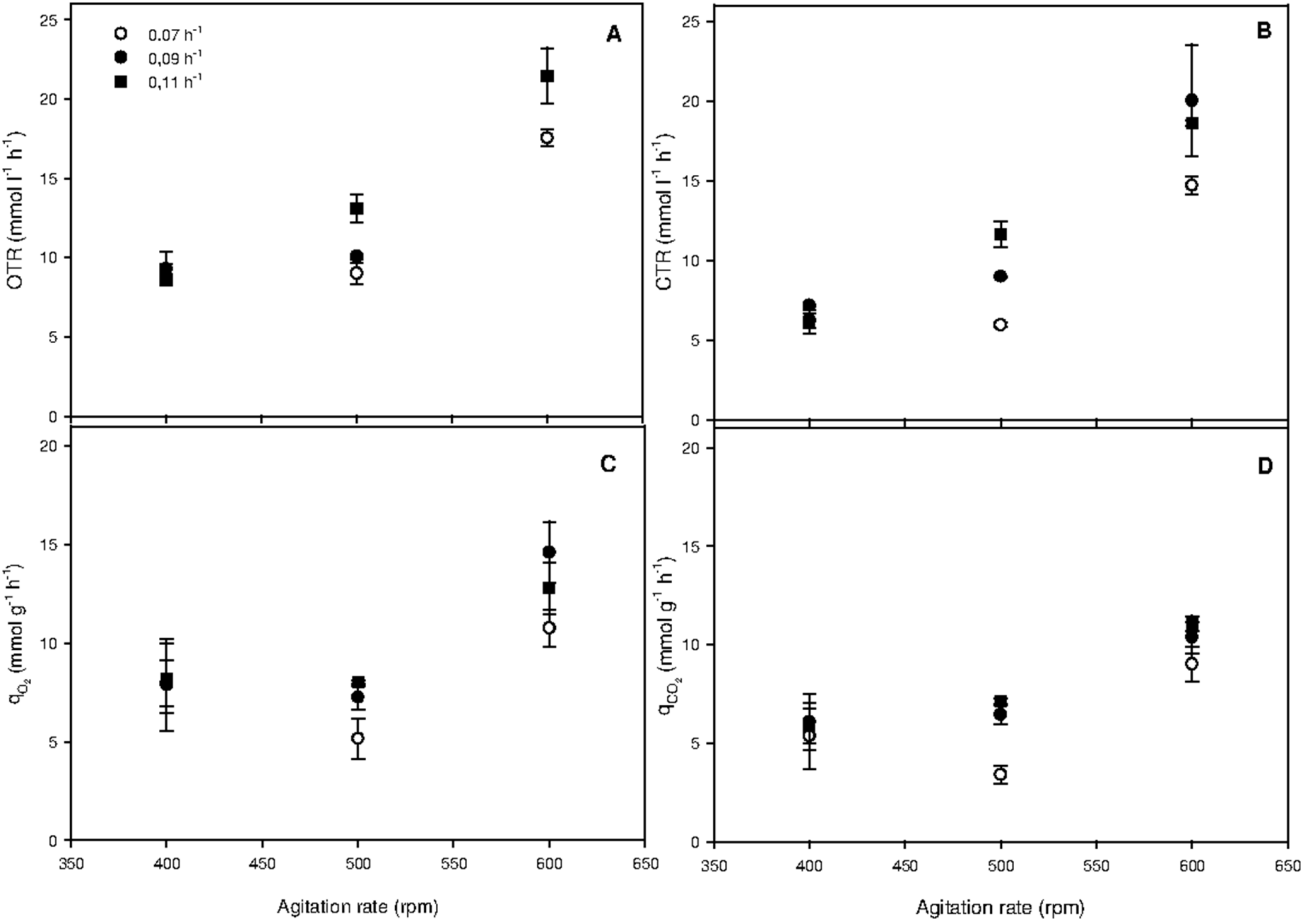
Respiratory activities of *A*. *vinelandii* as a function of the agitation rate and the dilution rate in continuous cultures. Data are reported as the mean value ± standard deviation.

Figure 2 shows the OTR, CTR, q_o2_ and q_co2_ obtained for the different chemostat cultures. At the lower agitation rate (400 rpm), a change in D did not affect the respiratory activity, as similar values of OTR, CTR, q_o2_ and q_co2_ were observed at the different dilution rates evaluated. However, at higher agitation rates (500 and 600 rpm), the respiratory activity was affected by D, demonstrating that the influence of D on respiratory activities is dependent on the imposed agitation rate.

As expected, an increase in agitation rate, particularly from 500 to 600 rpm, affected the OTR and q_o2_ level between 2 and 3-fold, depending on the D (Fig. 2a). OTR is a dynamic parameter influenced by operational conditions and the oxygen uptake rate (OUR) [19]. Under oxygen-limited conditions and at steady-state, the OTR equals the OUR; thus, these results could be interpreted as the effect of oxygen availability on alginate production.

The higher respiratory activity (in particular, the higher OTR and CTR values) obtained with the chemostat at 600 rpm could be related to higher sucrose consumption, suggesting that the carbon flux to the tricarboxylic acid (TCA) cycle is affected by changes in agitation rate or increased oxygen availability. In agreement with this observation, Castillo *et al*. [7] evaluated carbon flux using metabolic flux analysis and demonstrated increased flux through the TCA cycle in cultures of *A*. *vinelandii* grown under high aeration.

Table 2 shows the sucrose yields and sucrose consumption obtained for chemostat cultures at different D and agitation rates. Depending on the D and agitation rate, the sucrose yield on biomass (Y_x/s_) varied between 0.23 and 0.32 g g^−1^. Comparing the different dilution rates that were tested, the lowest Y_x/s_ (0.23 or 0.25 g g^−1^) was obtained in the chemostat agitated at 600 rpm. At 600 rpm, a higher CTR and q_co2_ were obtained (Fig. 2b, d), which may explain the lower Y_x/s_ obtained under these conditions. A decrease in the sucrose yield on alginate (Y_p/s_) was observed when D was increased. For example, in the chemostat agitated at 500 rpm, Y_p/s_ varied between 0.27 to 0.09 g g^−1^ when D was increased from D from 0.07 to 0.11 h^−1^. It is known that metabolism and physiology in a nutrient-limited state depend on the growth rate [17]; hence, the effect of D on Y_p/s_ could be related changes in carbon flux that allow a higher proportion of sucrose to be diverted to alginate.

**Table 2:** Sucrose yields and specific sucrose uptake rate for chemostat cultures of *A*. *vinelandii* at different D and agitation rates.

D (h^−1^)	Agitationrate (rpm)	Y_x/s_ (g g^−1^)	Y_p/s_ (g g^−1^)	q_s_ (g g^−1 ^h^−1^)
**0.07**	400	0.28±0.05	0.18±0.01	0.255±0.045
	500	0.27±0.02	0.27±0.01	0.257±0.014
	600	0.23±0.01	0.17±0.01	0.303±0.010
**0.09**	400	0.29±0.03	0.19±0.01	0.312±0.040
	500	0.27±0.03	0.13±0.01	0.333±0.041
	600	0.25±0.02	0.06±0.02	0.365±0.026
**0.11**	400	0.32±0.05	0.15±0.03	0.350±0.062
	500	0.31±0.01	0.09±0.01	0.353±0.002
	600	0.23±0.01	0.09±0.01	0.472±0.013

Values are means of the measurements ± SD.

As shown in Table 2, q_s_ varied between 0.26 to 0.47 g g^−1 ^h^−1^ at the different steady-states evaluated; these values are lower than the values previously reported by Díaz-Barrera *et al*. [8]. Those authors found values of q_s_ from 0.42 to 3.19 g g^−1 ^h in chemostat cultures conducted using an inlet sucrose concentration of 20 g l^−1^. In our work, an inlet sucrose concentration of 10 g l^−1^ was used, and it is possible that the differences in q_s_ could be caused by changes in cell metabolism due the differences in the sucrose consumption. Regardless of the D, an increase in agitation rate increased q_s_, indicating a variation in carbon source assimilation. To evaluate how carbon is distributed during continuous cultures, a carbon balance at each steady-state was performed.

### Carbon balances at steady-state

**Figure 3:**
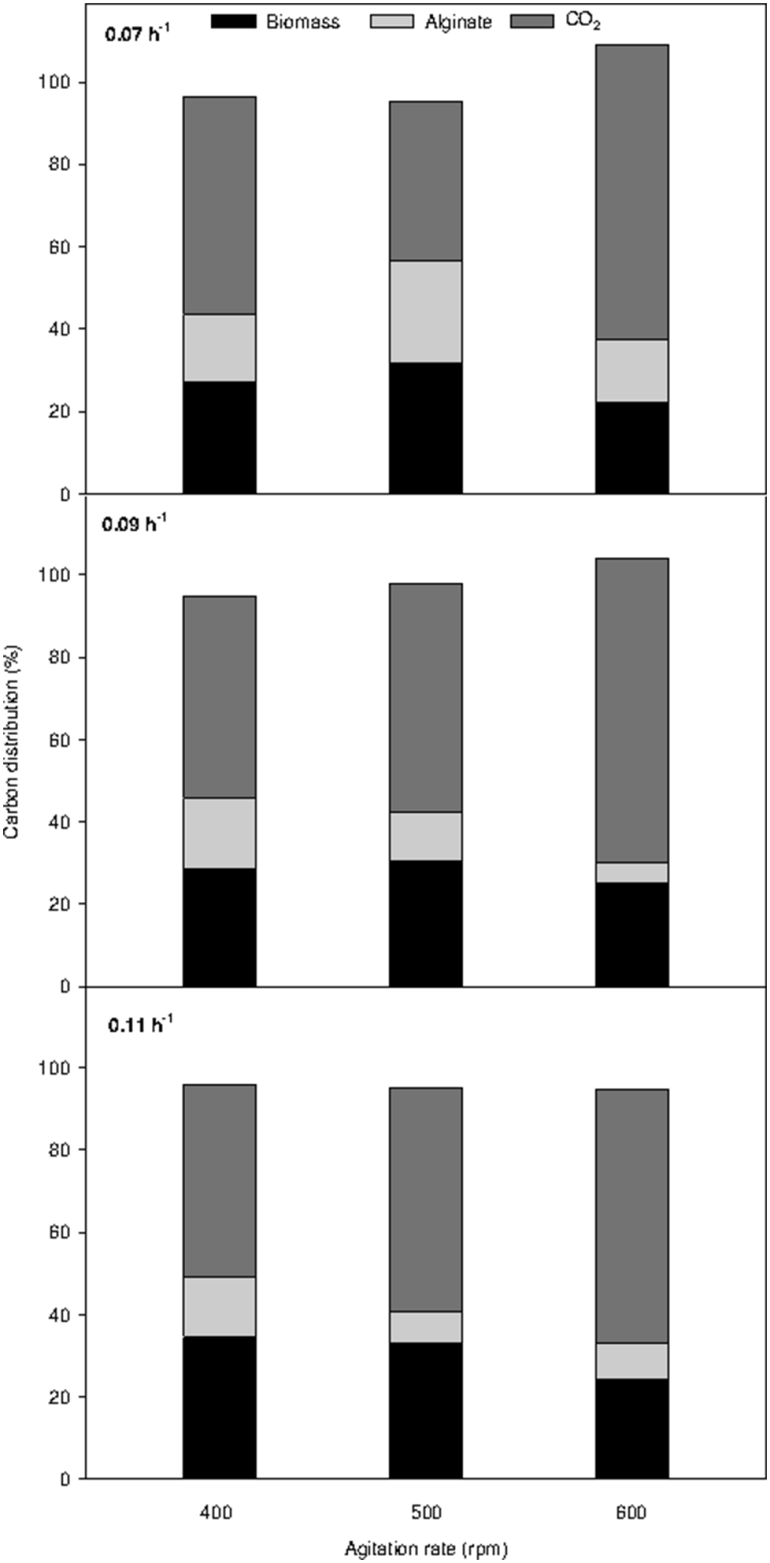
Carbon distribution for biomass, alginate, and carbon dioxide at different agitation rates in chemostat cultures of *A. vinelandii* conducted at 0.07, 0.09 and 0.11 h^−1^.

Carbon distribution, defined as the percentage of carbon atoms from sucrose converted to alginate, biomass (with PHB), and CO_2_ at each steady state condition, is presented as a function of agitation rate and D in Figure 3. The effect of D on carbon distribution to alginate depended on the agitation rate: in the chemostat operated at 500 rpm, an increase in D decreased the carbon diverted to alginate (from 25 to 7.9%). On the other hand, when the agitation rate was 400 rpm, carbon distribution to alginate was not significantly affected by changing D, remaining between 15 and 17%.

A higher carbon utilization for alginate synthesis was observed in the chemostat agitated at 500 rpm and 0.07 h^−1^ (25%), whereas a lower carbon utilization to alginate (5%) was obtained in cultures operated at 600 rpm and 0.09 h^−1^. This finding confirms that, to divert more carbon to alginate, it is necessary to operate chemostat cultures at an intermediate agitation rate and low D. In contrast, the carbon diverted to CO_2_ was greatly influenced by agitation rate in comparison to D. In chemostats with dilution rates of 0.07 and 0.09 h^−1^, increasing the agitation rate from 400 to 600 rpm increased the carbon diverted to CO_2_ from 50% to over 71%. In disagreement with this evidence, Díaz-Barrera *et al*. [9] demonstrated that when the agitation rate increased from 300 to 500 rpm, the percentage of carbon to CO_2_ remained almost unchanged, reaching approximately 50%. The different trend in carbon diverted to alginate and CO_2_ as a function of agitation rate observed in our study compared to Díaz-Barrera *et al*. [9] could be explained by the different carbon sources used. Considering these results, it is clear that both D and agitation rate affect the partitioning of the carbon flux into alginate and CO_2_ under oxygen-limited conditions.

At the different steady-states, the carbon balances closed to within 95–104%, suggesting that *A*. *vinelandii* cells utilized sucrose efficiently to produce alginate, CO_2_ and biomass (including PHB). Other carbon products were not produced, which was confirmed by measurements of organic acids using HPLC (data not shown). In disagreement with these results, Díaz-Barrera *et al*. [10] reported that in chemostat cultures fed sucrose (15 g l^−1^), a percentage of carbon (20 and 35%) was diverted to acetate and malate, which were released into the culture medium. This different behavior could be attributed to the non-nitrogen-fixation conditions used by Díaz-Barrera *et al*. [10], compared to the nitrogen-fixing conditions used in this work. In light of this observation, further research must be carried out to evaluate how nitrogen fixation conditions affect metabolic fluxes in *A*. *vinelandii* cultures.

Given that a higher proportion of carbon was diverted to alginate in the chemostat operated at 0.07 h^−1^ (Fig. 3), gene expression (*alg8*, *alg44, algL*, *alyA1*, *alyA2*, and *algE7*) and alginate production (in terms of molecular weight and production rate) were evaluated at the different agitation rates explored.

### Gene expression and alginate molecular weight at different agitation rates

**Figure 4:**
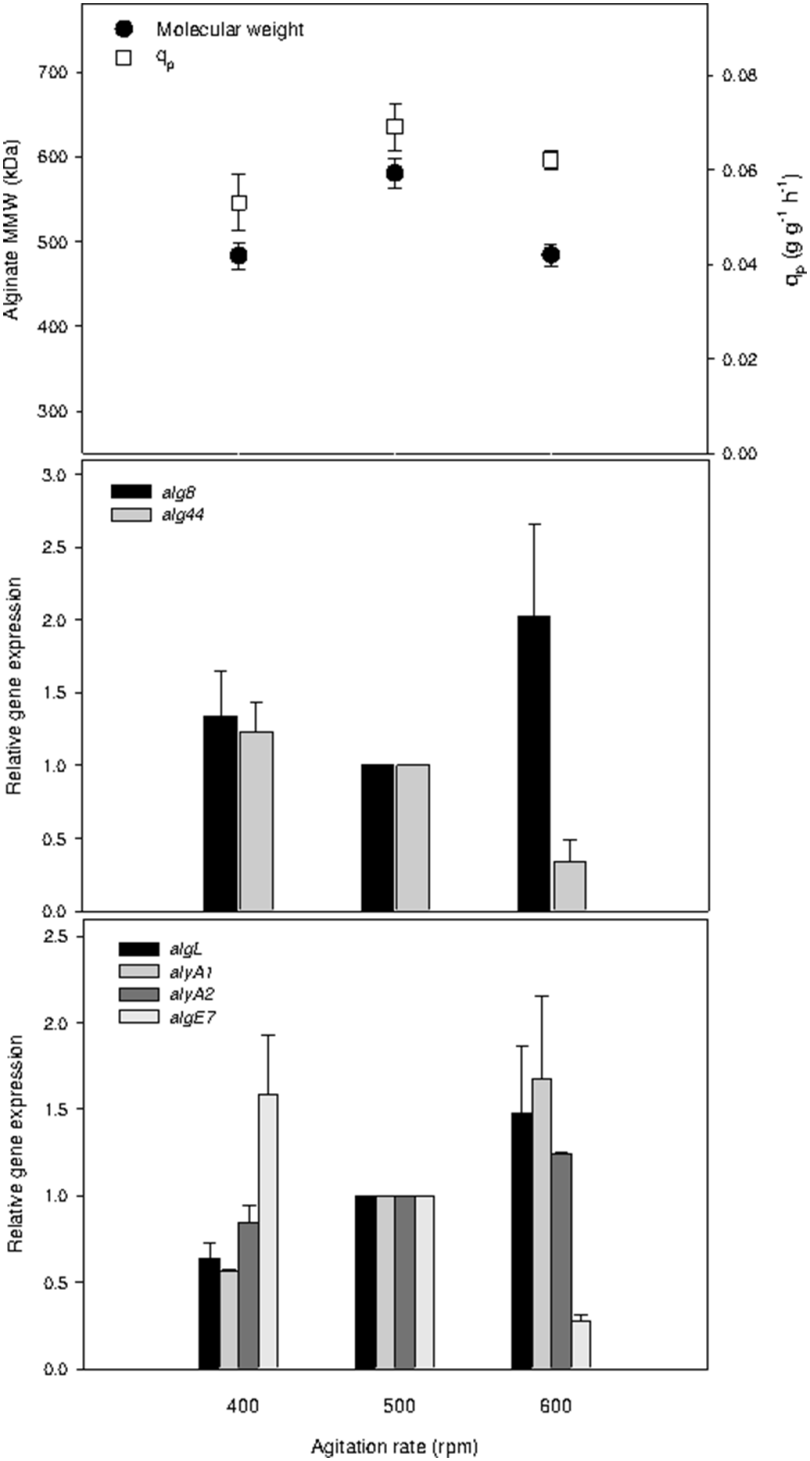
Effect of the agitation rate on alginate molecular weight, specific alginate production rate and expression of genes involved in alginate polymerization and depolymerization in chemostat cultures of *A. vinelandii* performed at a dilution rate of 0.07 h^−1^. The level of the *algL*, *alyA1, alyA2*, *algE7, alg8 and alg44* transcripts was normalized according to the level of the *gyrA* mRNA as described in Section Materials and Methods. The data are presented as fold changes respect of calibrator value (500 rpm). Each point is the mean ± standard deviation.

In previous reports, *algL* and *alg8* gene expression under different oxygen transfer rates (manipulated by agitation rate) in chemostat cultures of *A*. *vinelandii* has been studied [9], [10]. Recently, the expression of genes involved in polymerization and depolymerization of alginate under different DOT in batch cultures was also evaluated [11]. To complement the previous works performed at specific growth rate (i.e., in chemostat cultures), the alginate mean molecular weight (MMW), specific alginate production rate (q_p_), and gene expression of *algL*, *alyA1*, *alyA2*, *algE7*, *alg8* and *alg44* were evaluated at different agitation rates under nitrogen-fixation conditions in a continuous culture operated at 0.07 h^−1^ (Fig. 4).

A higher molecular weight was obtained in chemostat cultures agitated at 500 rpm (580 kDa), while a similar molecular weight (480 kDa) was observed for 400 and 600 rpm (Fig. 4a). A similar behavior was observed for q_p_ due to that values highest (0.069 g g^−1 ^h^−1^) were observed at 500 rpm, such as has been previously described [10]. As shown in Fig. 4b, it is clear that the expression of both *alg44* and *alg8* were affected by changes in agitation rate. The *alg44* expression decreased by a factor of approximately 3.6 when the agitation rate was increased from 400 to 600 rpm, while the expression of *alg8* was highest at 600 rpm compared to the other conditions evaluated. These data suggest that both *alg44* and *alg8* gene expression could be modulated by oxygen availability.

The highest alginate MMW was produced at 500 rpm (Fig. 4a); however, this result cannot be explained by the expression patterns of *alg8* or *alg44*. In disagreement with these results, Díaz-Barrera *et al*. [10] reported that a higher alginate MMW in chemostat cultures can be related to higher *alg8* gene expression, suggesting that in *A*. *vinelandii* cells, *alg8* encodes the proposed catalytic subunit of alginate polymerase. The difference in behaviors observed in this study and by Díaz-Barrera *et al*. [10] may be explained by physiological differences that result from the different culture conditions used: while Díaz-Barrera *et al*. [10] used non-nitrogen-fixation conditions, nitrogen fixation conditions were used in this work.

Previous studies of in-vitro polymerization showed that the entire cell envelope was required for alginate polymerization, suggesting that Alg8 requires other proteins to function [20]. Although the specific role that Alg44 plays in polymerization remains unclear, Alg44 could play an indirect role. It has been suggested that there is a mutual stability relationship between Alg8 and Alg44 [3]. The findings obtained in our study, which indicate that the expression of *alg8* and *alg44* was not related to higher alginate MMW (obtained at 500 rpm), could be explained by a mutual stability relationship between Alg8 and Alg44. Further studies related with enzymatic activity should be carried out to evaluate this possibility.

Gimmestad *et al*. [5] suggested that AlyA1 and AlyA2 are intracellular enzymes, and it is known that the periplasmic AlgL is intracellular whereas the alginate lyase AlgE7 is extracellular in *A*. *vinelandii* [21]. Interestingly, different expression patterns were observed for lyases (*algL, alyA1* and *alyA2*) and *algE7* in response to changes in agitation rate (and hence oxygen availability) at steady-state. A 10-fold decrease in *algE7* expression was observed when the agitation rate was increased from 400 to 600 rpm was observed, while an increase in lyase expression (2.8, 2.5 and 1.5 times for *alyA1*, *algL* and *alyA2*, respectively) was observed under the same conditions (Fig. 4c). The results of our work suggest that higher oxygen availability (determined by a higher agitation rate) affect lyase expression levels, increasing the expression of intracellular lyases when OTR level is increased.

Given that increased lyase (*algL*, *alyA1* and *alyA2*) expression was observed when the agitation rate was increased, the similar alginate MMW values obtained (480 kDa) at 400 and 600 rpm cannot be explained by changes in the expression levels of intracellular lyases. It is possible that at the lower agitation rate (400 rpm), higher expression of *algE7* and, possibly, increased lyase activity could explain the similar alginate MMW obtained at 400 rpm compared to 600 rpm. Similarly, Flores *et al*. [11] recently observed higher expression of *algL* and *alyA2* as well as a high alginate MMW (1200 kDa) in batch cultures at lower DOT (1% compared to 5%). This contradictory behavior was explained by an analysis of alginase activity. Flores *et al*. [11] found a basal level of extracellular lyase activity in the batch cultures at 1% DOT, which is consistent with the high alginate MMW obtained. In light of the evidence obtained, it is possible that alginate molecular weight results from a balance between gene expression of intracellular and extracellular lyases in response to the agitation rate of the culture. To our knowledge, our findings for the first time show lyase expression as a function of oxygen availability at a constant growth rate. Furthermore, these evidences demonstrate how different expression patterns could determine the molecular weight of the alginate synthetized at steady-state.

### Conclusion

In this work, the effect of agitation rate on alginate production and the expression of genes involved in alginate polymerization and depolymerization were evaluated in continuous *A*. *vinelandii* cultures. Under oxygen-limited conditions, the agitation rate influenced the partitioning of carbon into alginate and CO_2_. In chemostat cultures performed at 0.07 h^−1^ and 500 rpm, a highest alginate molecular weight (580 kDa) as compared to 400 and 600 rpm was obtained. Different expression levels of lyase genes (intra and extracellular), modulated by the oxygen supply conditions, could explain the changes in the molecular weight, particularly the highest alginate molecular weight obtained at 500 rpm. The findings provide knowledge about alginate polymerization process in *A*. *vinelandii* and open up new possibilities of synthesizing polymers with particular molecular weight.
